# Integrating Genomic and Transcriptomic Data to Reveal Genetic Mechanisms Underlying Piao Chicken Rumpless Trait

**DOI:** 10.1016/j.gpb.2020.06.019

**Published:** 2021-02-23

**Authors:** Yun-Mei Wang, Saber Khederzadeh, Shi-Rong Li, Newton Otieno Otecko, David M. Irwin, Mukesh Thakur, Xiao-Die Ren, Ming-Shan Wang, Dong-Dong Wu, Ya-Ping Zhang

**Affiliations:** 1 State Key Laboratory of Genetic Resources and Evolution, Kunming Institute of Zoology, Chinese Academy of Sciences, Kunming 650223, China; 2 Kunming College of Life Science, University of the Chinese Academy of Sciences, Kunming 650223, China; 3 Center for Neurobiology and Brain Restoration, Skolkovo Institute of Science and Technology, Moscow 143026, Russia; 4 Department of Laboratory Medicine and Pathobiology, University of Toronto, Toronto M5S 1A8, Canada; 5 Zoological Survey of India, Kolkata 700053, India

**Keywords:** Comparative transcriptomics, Population genomics, Rumplessness, Vertebra development, Artificial selection

## Abstract

Piao chicken, a rare Chinese native poultry breed, lacks primary tail structures, such as pygostyle, caudal vertebra, uropygial gland, and tail feathers. So far, the molecular mechanisms underlying tail absence in this breed remain unclear. In this study, we comprehensively employed **comparative transcriptomic** and genomic analyses to unravel potential genetic underpinnings of **rumplessness** in Piao chicken. Our results reveal many biological factors involved in tail development and several genomic regions under strong positive selection in this breed. These regions contain candidate genes associated with rumplessness, including *Irx4*, *Il18*, *Hspb2*, and *Cryab*. Retrieval of quantitative trait loci (QTL) and gene functions implies that rumplessness might be consciously or unconsciously selected along with the high-yield traits in Piao chicken. We hypothesize that strong selection pressures on regulatory elements might lead to changes in gene activity in mesenchymal stem cells of the tail bud. The ectopic activity could eventually result in tail truncation by impeding differentiation and proliferation of the stem cells. Our study provides fundamental insights into early initiation and genetic basis of the rumpless phenotype in Piao chicken.

## Introduction

Body elongation along the anterior–posterior axis is a distinct phenomenon during vertebrate embryo development. Morphogenesis of caudal structures occurs during posterior axis elongation. The tail bud contributes most of the tail portion [1]. This structure represents the remains of the primitive streak and Hensen’s node, and comprises a dense mass of undifferentiated mesenchymal cells [1]. Improper patterning of the tail bud may give rise to a truncated or even absent tail [2]. Previous investigations implicated many factors involved in the formation of posterior structures. For example, loss of *T Brachyury Transcription Factor* (*T*) causes severe defects in mouse caudal structures, including the lack of the notochord and allantois, abnormal somites, and a short tail [3]. Genetic mechanisms for rumplessness vary among the different breeds of rumpless chickens. For instance, Wang et al. revealed that rumplessness in Hongshan chicken, a Chinese indigenous breed, is a Z chromosome-linked dominant trait and may be associated with the region containing candidates like *Lingo2* and the pseudogene *LOC431648* [4], [5]. In Araucana chicken, a Chilean rumpless breed, the rumpless phenotype is autosomal dominant and probably related to two proneural genes — *Irx1* and *Irx2* [6], [7].

Piao chicken, a Chinese autochthonic rumpless breed, is native to Zhenyuan County, Puer City, Yunnan Province, China, and is mainly found in Zhenyuan and adjacent counties [8]. This breed has no pygostyle, caudal vertebra, uropygial gland, and tail feathers [8], and hence is an ideal model for studying tail development [9]. Through crossbreeding experiments and anatomical observations, Song et al. showed that rumplessness in Piao chicken is autosomal dominant and forms during the embryonic period, although no specific stage was identified [9], [10]. However, until now, the genetic mechanisms of rumplessness in this breed have not yet been elucidated.

Advances in next-generation sequencing and microscopy have made it possible to probe embryonic morphogenesis through microscopic examination, to study phenotypic evolution using comparative population genomics, and to assess transcriptional profiles associated with specific characteristics via comparative transcriptomics. In this study, we integrated the three methods to uncover the potential genetic basis of the rumpless phenotype in Piao chicken.

## Results

### Comparative genomic analysis identifies candidate regions for the rumpless trait in Piao chicken

**Figure 1:**
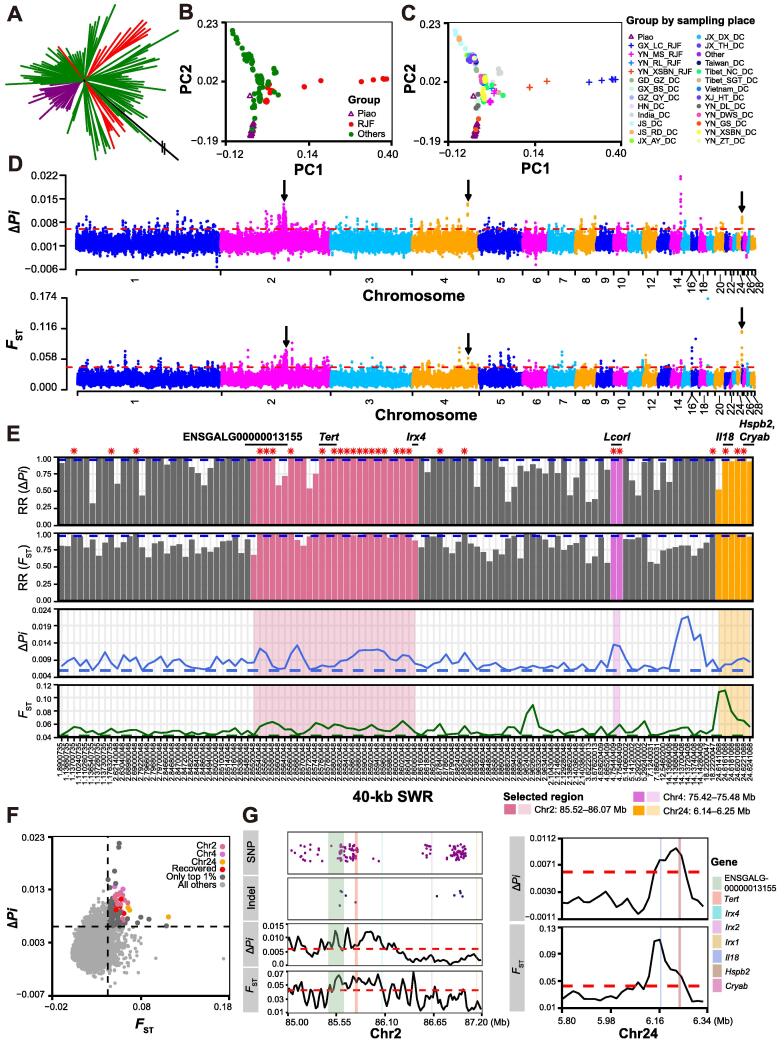
**Population genomic analysis** **A.** Weighted phylogenetic tree of Piao chickens (purple) and controls grouped into RJF (red), GJF outgroup (black), and others (green). The double slash represents the outside part not shown in the figure. **B.** PCA plot of all samples except GJF. Groups are colored as in (A). Empty triangles mark Piao chickens, while solid points are controls. **C.** PCA plot similar to (B) but with controls from different places marked by different colors and shapes. Crosses represent RJFs, and solid points represent other domestic chickens. GX_BS_DC, domestic chicken from Baise, Guangxi; YN_DL_DC, domestic chicken from Dali, Yunnan; YN_GS_DC, domestic chicken from Gongshan, Yunnan; for the specification of other abbreviations please see Table S1. **D.** Manhattan plots of *F*_ST_ and Δ*Pi* of 40-kb SWRs. Black arrows point out the three strongly selected regions mentioned in the main text. **E.***F*_ST_ and Δ*Pi* values (the bottom two line charts with dashed lines indicating the top 1% threshold), and their random sampling RRs (the top two bar plots with dashed lines indicating RR = 0.95) for the 112 selected 40-kb SWRs. Red asterisks mean RR ≥ 0.95 for both *F*_ST_ and Δ*Pi*. Candidate genes are indicated using short black lines. Color fillers represent the 40-kb SWRs intersecting with the three strongly selected regions. **F.** Scatter plot for *F*_ST_ and Δ*Pi* of 40-kb SWRs. Larger dots are the 112 selected 40-kb SWRs colored as in (E) if RR ≥ 0.95. **G.** Line charts of *F*_ST_ and Δ*Pi* values around chr2: 85.52–86.07 Mb (left) and chr24: 6.14–6.25 Mb (right). Dashed lines indicate the top 1% threshold. Dark magenta and midnight blue dots represent highly differentiated SNPs and indels, respectively. Color fillers are genes located in the two regions. All 40-kb SWRs are shown by their median sites. RJF, red junglefowl; GJF, green junglefowl; PCA, principal component analysis; SWR, sliding window region; *F*_ST_, fixation index; Δ*Pi*, nucleotide diversity; RR, recovery ratio; SNP, single-nucleotide polymorphism; indel, insertion/deletion.

Using comparative population genomics, we evaluated the population differentiation between the Piao chicken and control chickens with a normal tail. In total, we analyzed the genomes of 20 Piao and 98 control chickens, which included 18 red junglefowls (RJFs), 79 other domestic chickens, and 1 green junglefowl (GJF; as an outgroup) (Table S1). We identified 27,557,576 single-nucleotide polymorphisms (SNPs), with more than half (52.5%) in intergenic regions, 42.1% in introns, and 1.5% in exons. Functional annotation by ANNOVAR [11] revealed that 292,570 SNPs (∼ 1%) cause synonymous substitutions, while 131,652 SNPs (∼ 0.5%) may alter protein structures and functions through non-synonymous mutations or gain and loss of stop codons. A phylogenetic tree and principal component analysis (PCA) showed certain population differences between the Piao chicken and controls (Figure 1A–C). High genetic relatedness was found between the Piao and domestic chickens from Gongshan, Yunnan (YN_GS_DC; Figure 1C). One of the Piao chicken samples clearly separated from the other Piao chickens and was close to the Chinese domestic chickens from Dali, Yunnan (YN_DL_DC) and Baise, Guangxi (GX_BS_DC). Since Zhenyuan, Dali, and Baise are geographically close neighbors, it is possible that Piao chicken is beginning to mix with these exotic breeds due to emerging advances in the transport networks in these regions.

Based on fixation index (*F*_ST_), nucleotide diversity (Δ*Pi*), and genotype frequency (GF), we searched for genomic regions, SNPs, and insertions/deletions (indels) that had high population differences between the Piao and control chickens (see Materials and methods). We identified 112 40-kb sliding window regions (SWRs) with strong signals of positive selection in Piao chicken, out of which 28 were highly recovered in 1000 random samples of 20 controls from the 98 control chickens (Figure 1D–G; Table S2). The three regions with the strongest repeatable selection signals were chr2: 85.52–86.07 Mb, chr4: 75.42–75.48 Mb, and chr24: 6.14–6.25 Mb.

Chr2: 85.52–86.07 Mb exhibited a high proportion of highly differentiated SNPs and indels (GF > 0.8), approximately 15.6% (76/488) and 10.4% (5/48), respectively (Figure 1G; Table S2). There are many important genes in this region. For instance, *Irx4*, along with *Irx1* and *Irx2*, belongs to the *Iroquois* genomic cluster — *IrxA* [12]. Tena et al. discovered an evolutionarily conserved three-dimensional structure in the vertebrate *IrxA* cluster, which facilitates enhancer sharing and coregulation [12]. *Irx4* was reported to be involved in the differentiation of progenitor cells [13], [14]. Although *Irx4* itself was out of the 28 highly recovered 40-kb SWRs, its intergenic region with *Ndufs6* was largely covered. Another gene in this region, *Tert*, is implicated in spermatogenesis and male infertility [15]. In particular, ENSGALG00000013155, a novel gene, showed strong selection signals from both *F*_ST_ and Δ*Pi*. This gene had 39 highly differentiated SNPs (23 located in introns and 16 in the intergenic region with *Mocos*) and two highly differentiated indels (in introns). We retrieved its expression profiles in different chicken tissues and development stages from three NCBI projects (SRA: ERP003988, SRP007412, and DRP000595) used in our previous study [16] (Figure S1A). The results showed that this gene was highly expressed in adipose, adrenal gland, and cerebellum. Its expression also gradually increased during the early development of chicken embryo. The adrenal gland affects lipid metabolism and sexual behavior [17], [18], while the cerebellum is involved in emotional processing [19]. These results suggest that ENSGALG00000013155 plays important roles in fat deposition, sexual behavior, and embryogenesis in chicken.

Chr4: 75.42–75.48 Mb includes one gene — *Lcorl*. *Lcorl* was reported as a candidate gene associated with the internal organ weights in chicken [20], body size in horse [21], and production performances in cattle [22].

Chr24: 6.14–6.25 Mb exhibited the strongest selection signal on chromosome 24 and has a fundamental gene — *Il18* (Figure 1G). *Il18* is well known as a proinflammatory and proatherogenic cytokine, as well as an IFN-γ inducing factor [23]. The gene modulates acute graft-*versus*-host disease by enhancing Fas-mediated apoptosis of donor T cells [24]. It can also induce endothelial cell apoptosis during myocardial inflammation and injury [25]. Interestingly, this gene has been reported to play prominent roles in osteoblastic and osteoclastic functions that are crucial for bone remodeling by balancing formation and resorption [26], [27]. *Il18* is also known as a trigger of nuclear factor-κB (NF-κB) activation [25]. Sequestration of NF-κB in zebrafish can disrupt the notochord differentiation and bring about *no tail* (*ntl*)-like embryos [28]. This no tail phenotype can be rescued by the T-box gene *ntl* (*Brachyury* homologue) [28]. Indeed, in chicken, blockage of NF-κB expression in limb buds gives rise to a dysmorphic apical ectodermal ridge, a decrease in limb size and outgrowth, and a failure of distal structures, through the interruption of mesenchymal–epithelial communication [29].

### Developmental transcriptome analysis reveals differentially expressed genes associated with tail development

The tail bud begins to form at the Hamburger-Hamilton stage 11 of chicken embryo development [30], [31], and undergoes multidimensional morphogenesis in the subsequent three to ten days [32]. Thus, it should be possible to analyze transcriptional diversity associated with tail generation during this period. Based on this premise, we sought to outline the functional factors involved in caudal patterning using RNA sequencing data from 9 Piao and 12 control chicken embryos after seven to nine days (D7–D9) of incubation (Table S1; see Materials and methods).

**Figure 2:**
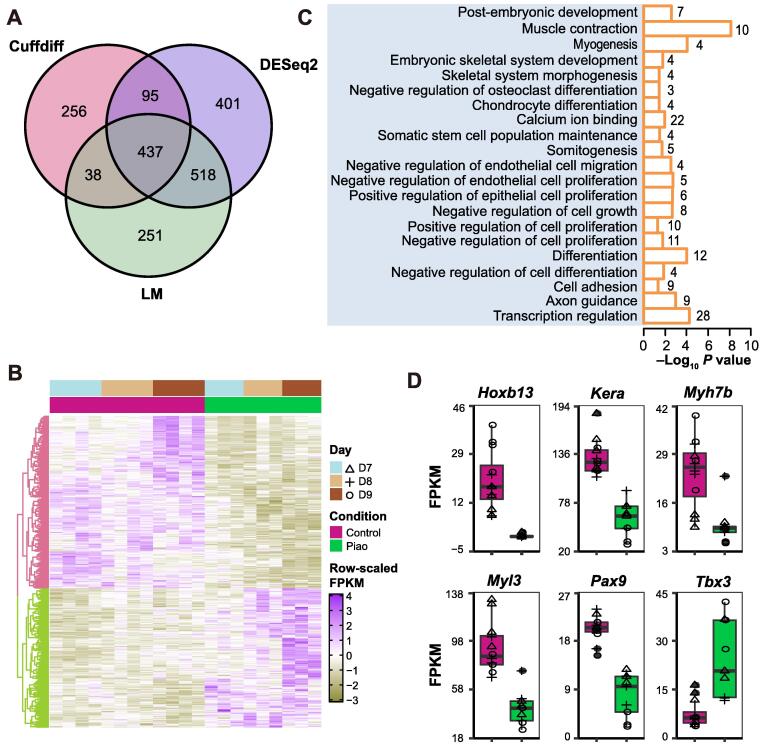
**Differential expression analysis** **A.** Relationships of the numbers of DEGs from Cuffdiff, DESeq2, and LM. **B.** Heatmap and hierarchical clustering dendrogram based on the FPKM expression values of DEGs. Rows represent DEGs, while columns show samples. **C.** Significant categories of functional annotation among DEGs. Digit on the right of a bar represents gene number in the category. **D.** FPKM values of six representative DEGs in Piao and control chickens across development days marked by shapes as shown in (B). DEG, differentially expressed gene; LM, linear model; FPKM, fragments per kilobase of exon model per million reads mapped.

Evaluation of expression patterns identified 437 differentially expressed genes (DEGs) between the Piao and control chicken embryos across D7–D9 (Figure 2A and B; Table S3), including the gene *T* that is implicated in tail development [3]. Functional enrichment analysis of the DEGs by the database for annotation, visualization and integrated discovery (DAVID; v6.8) [33] showed that many biological processes were related to posterior patterning, including muscle development, bone morphogenesis, and somitogenesis, as well as cell differentiation, proliferation, and migration (Figure 2C).

Some interesting DEGs had expression fold changes greater than 2 between Piao and control chickens, including *Tbx3*, *Hoxb13*, *Myl3*, *Myh7b*, *Kera*, and *Pax9* (Figure 2D). *Tbx3*, encoding a transcription factor, had 2-fold higher expression in Piao chickens than in controls. This gene is known to regulate osteoblast proliferation and differentiation [34]. *Hoxb13*, located at the 5′ end of the *HOXB* cluster, had almost undetectable expression in Piao chickens, but 35.7-fold higher level in controls. In mice, loss-of-function mutations in *Hoxb13* cause overgrowth of posterior structures by disturbing proliferation inhibition and apoptosis activation [35]. *Myl3* showed 1.2-fold lower expression in Piao chickens compared to controls. This gene encodes a myosin alkali light chain in slow skeletal muscle fibers and modulates contractile velocity [36]. *Myh7b*, with 1.4-fold lower expression in Piao chickens, encodes the third myosin heavy chain [37]. Mutations in *Myh7b* cause a classical phenotype of left ventricular non-compaction cardiomyopathy [37]. *Kera* encodes an extracellular matrix keratocan, which acts as an osteoblast marker regulating osteogenic differentiation [38]. *Kera* was expressed 1.3-fold lower in Piao chickens than in controls. *Pax9* and *Pax1* function redundantly to influence the vertebral column development [39]. Compared to the controls, the expression level of *Pax9* in Piao chickens is nearly 1.6-fold lower. Analyzing these gene expression patterns revealed that multiple genes are likely involved in chicken tail development.

### Co-expression modules delineate the biological processes relevant to posterior patterning

**Figure 3:**
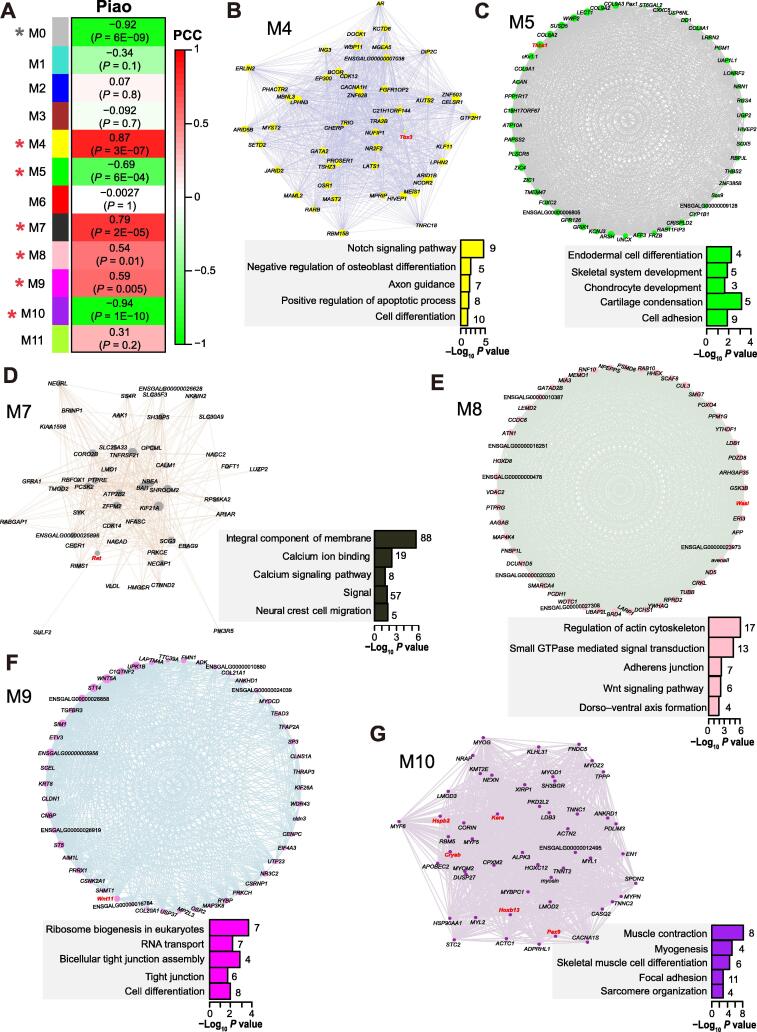
**Gene co-expression module analysis** **A.** Module relationships with rumplessness. Value outside of the parenthesis indicate PCC. Modules are gradiently colored by PCC. Modules significantly correlated to rumplessness are marked with a red asterisk, while a gray asterisk labels M0 to indicate that the module was excluded from the further analyses. **B.**–**G.** Network plots based on the top 50 hub genes of the six significant modules. Dot size indicates gene connectivity to others in the module. The hub genes mentioned in the main text were marked in red. Significant annotation categories of these modules are colored in bars according to the network dots. The number on the right of a bar presents gene number in the category. PCC, Pearson correlation coefficient.

To further elucidate the principal biological pathways regarding caudal development, we constructed correlation networks through a weighted gene co-expression network analysis (WGCNA) [40] (see Materials and methods). We captured twelve co-expression modules, six of which (M4, M5, M7, M8, M9, and M10) were significantly correlated with the rumpless phenotype (Pearson correlation, *P* < 0.05) (Figure 3A–G).

Functional annotation of the eigengenes in these significant modules revealed close linkages with embryonic development. The negatively correlated modules M5 and M10 showed functional enrichment for skeletal system development and myogenesis, respectively (Figure 3C and G). Modules in positive correlation with rumplessness were implicated in several different pathways: axon guidance and osteoblast differentiation for M4; calcium signaling pathway and neural crest cell migration for M7; actin cytoskeleton organization and dorso–ventral axis formation for M8; transcription and tight junction for M9 (Figure 3B and D–F).

We searched for hub genes in each significant module (Figure 3B–G; Table S4). Interestingly, *Tbx3*, one of the six DEGs mentioned above, was an M4 hub gene. Another five DEGs (*Hoxb13, Myl3*, *Myh7b*, *Kera*, and *Pax9*) were all M10 hub genes. The M7 hub gene *Ret* encodes a transmembrane tyrosine kinase receptor with an extracellular cadherin domain [41]. *Ret* induces enteric neuroblast apoptosis through caspase-mediated self-cleavage [42]. The M5 hub gene *Thbs1* affects epithelial-to-mesenchymal transition and osteoporosis [43], [44]. An M8 hub gene *Wasl* is essential for Schwann cell cytoskeletal dynamics and myelination [45]. The M9 hub gene *Wnt11* is crucial for gastrulation and axis formation [46], [47]. These findings suggest that functional polygenic inter-linkages influence posterior patterning during chicken development.

### Two DEGs under strong selection co-localized with *Il18*

To determine whether our DEGs were strongly selected, we retrieved genes in the 40-kb SWRs with strong repeatable signals of selection. We simultaneously searched for any highly differentiated SNP and indel in or flanking these DEGs. Only two DEGs under strong selection were identified, *i.e.*, *Hspb2* and *Cryab*. Unfortunately, we found no highly differentiated SNP or indel related to these two DEGs. *Hspb2* and *Cryab* are both located near *Il18* in chr24: 6.14–6.25 Mb (Figure 1G). They encode small heat shock proteins that are essential for calcium uptake in myocyte mitochondria [48]. Nevertheless, they function non-redundantly: *Hspb2* balances energy as a binding partner of dystrophin myotonic protein kinase, while *Cryab* is implicated in anti-apoptosis and cytoskeletal remodeling [49].

## Discussion

Accurate molecular regulation and control are vital for biological development and existence. Interfering these functional networks can lead to embryo death, diseases, deformities, or even the evolution of new characters [3], [4], [5], [6], [7]. Selection makes domestic animals achieve numerous phenotypic changes in morphology, physiology, or behavior by modulating one or more components of primary biological networks [16].

**Figure 4:**
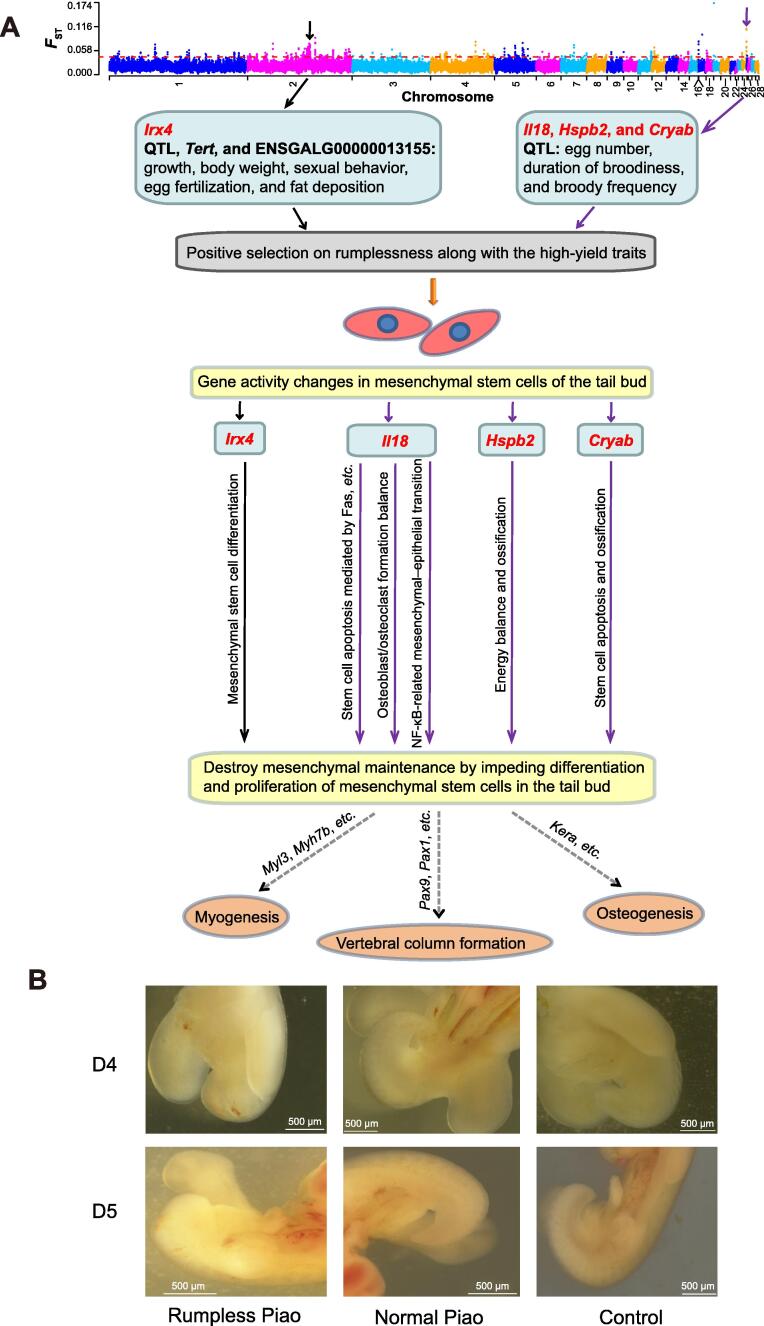
**Proposed mechanisms underlying rumplessness in Piao chicken and microscopic examination of chicken tail embryogenesis** **A.** Rumplessness might be consciously or unconsciously selected along with the high-yield traits of Piao chicken. Strong positive selection pressures on regulatory elements of some candidate genes might lead to gene activity changes in the tail bud. The ectopic expression of these genes would destroy mesenchymal maintenance by impeding differentiation and proliferation of mesenchymal stem cells in the tail bud, through multiple cell survival and differentiation pathways. This could then disrupt tail formation and prevent later developmental processes of the distal structures. **B.** The morphology of the posterior regions on D4 and D5 in rumpless Piao chicken, Piao chicken with a normal tail, and a control chicken. Scale bar, 500 µm. QTL, quantitative trait loci.

In this study, we combined comparative transcriptomics and population genomics to explore the genetic mechanisms underlying rumplessness of Piao chicken. Our transcriptomic analyses identified many biological pathways that might be important to the late development of chicken tail. Genome-wide comparative analyses revealed several genomic regions under robust positive selection in Piao chicken. These regions contain some fundamental genes, including *Tert*, ENSGALG00000013155, *Irx4*, *Lcorl*, *Il18*, *Hspb2*, and *Cryab*. Only *Hspb2* and *Cryab* were DEGs between the Piao and control chickens during D7–D9. Some of these genes might be associated with performance traits in Piao chicken, such as *Tert* for egg fertilization, ENSGALG00000013155 for fat deposition, and *Lcorl* for body weight. Others might be implicated in axis elongation, such as *Irx4*, *Il18*, *Hspb2*, and *Cryab*, through signaling pathways like NF-κB, calcium, or apoptosis. Meanwhile, by retrieving all available quantitative trait loci (QTL) from the Animal QTLdb [50], we found that these selected regions were associated with production traits, such as growth, body weight, egg number, duration of broodiness, and broody frequency (Table S2). Despite the evolutionary history of Piao chicken being unclear, this breed is characterized by high production, including elevated fat deposition rates, meat production, egg fertilization, and egg hatchability [8], [51]. Piao chicken originates in a relatively isolated environment and has limited genetic admixture with exotic breeds [51]. Nevertheless, the breed has high genetic variability and five maternal lineages [52], implying its high hybrid fertility. Thus, we speculate that rumplessness, which exposes the posterior orifice of Piao chicken, might make intra-population or inter-population mating easier for the breed. This easier mating might increase egg fertilization, egg number, broody frequency, and genetic variability in Piao chicken. We propose that rumplessness might be consciously or unconsciously selected along with the high-yield traits of Piao chicken (Figure 4A).

In addition, we found that all highly differentiated SNPs and indels, related to the aforementioned candidate genes, are located in non-coding regions or cause synonymous mutations. Therefore, we hypothesize that positive selection pressures on the regulatory elements of some of these candidate genes would produce functional changes, which then leads to the rumpless phenotype in Piao chicken (Figure 4A). These activity changes likely occur at very early development stages. In fact, when examining embryogenesis, we observed extreme tail truncation in most of the Piao embryos after D3, while some Piao and all control embryos displayed normal posterior structures (Figure 4B). The caudal morphogenesis in Piao embryos confirmed the descriptions by Song et al. [9] and Zwilling [53]. Song et al. found that the rumpless phenotype in Piao chicken is autosomal dominant [9]. Zwilling stated that dominant rumpless mutants arise at the end of D2 and are established by the closure of D4 [53]. Previous studies have shown that the tail bud comprises a dense mass of undifferentiated mesenchymal cells and forms most of the tail portion [1]. This structure undergoes multidimensional morphogenesis from D3 [32]. Thus, we postulate that ectopic expression, driven by positive selection pressures, might be initiated in the tail bud and impede normal differentiation and proliferation of the mesenchymal stem cells through signaling pathways like NF-κB, calcium, or proapoptosis (Figure 4A). The result is the derailing of mesenchymal maintenance in the tail bud and the eventual failure of normal tail development. Hindrance to tail formation might have overarching impacts on later developmental processes of distal structures, for instance, *Myl3* and *Myh7b* involved in myogenesis, or *Pax9* and *Pax1* participating in vertebral column formation (Figure 4A). In particular, the strong selection on the proinflammatory cytokine gene *Il18* could also be an adaptive response for robust immunity, as rumplessness leaves the posterior orifice exposed to infections. However, as an autosomal dominant phenotype expressed very early in development, it is hard to confirm rumplessness in embryos and samples without RNA degradation before D4. Therefore, we could barely even validate the expression of the identified genes in the tail bud further.

Different genetic architectures of taillessness have been found among different chicken breeds [4], [5], [6], [7], *e.g.*, Hongshan chicken [4], [5] *versus* Araucana chicken [6], [7]. Interestingly, Hongshan chicken has normal coccygeal vertebrae and its rumpless trait is Z-linked dominant, while Araucana and Piao breeds have no caudal vertebrae and their rumplessness is autosomal dominant. Our results also reveal potential spatiotemporal similarities between Piao and other autosomal-dominant rumpless chickens [6], [7], [53]. This implies that the autosomal-dominant rumpless trait in chicken probably has the same genetic mechanisms and embryogenesis. Overall, this study provides a basic understanding of the genes and biological pathways that may be related, directly or indirectly, to rumplessness in Piao chicken. Our work could shed light on tail degeneration in vertebrates. Future endeavors should address the limitation to discern specific causative mutations that lead to tail absence in chicken.

## Conclusion

By combining comparative transcriptomics, population genomics, and microscopic examination of embryogenesis, we reveal the potential genetic mechanisms of rumplessness in Piao chicken. This work could facilitate a deeper understanding of tail degeneration in vertebrates.

## Materials and methods

### Whole-genome re-sequencing data preparation

DNA was extracted from the blood samples of 20 Piao chickens by the conventional phenol–chloroform method. Quality check and quantification were performed using agarose gel electrophoresis and NanoDrop 2000 spectrophotometer. Paired-end libraries were prepared by the NEBNext Ultra DNA Library Prep Kit for Illumina (Catalog No. E7370, NEB, Ipswich, MA) and sequenced on the Illumina HiSeq2500 platform after quantification. 150-bp paired-end reads were generated. We obtained ten-fold average sequencing depth for each individual. For controls, we used genomes of 96 chickens with a normal tail from an unpublished project in our laboratory, including one GJF outgroup, 18 RJFs, and 77 other domestic chickens (Table S1). Sequence coverage for these genomes ranged from about 5 to 110. Control genomic data from two additional individuals were downloaded from NCBI (SRA: SRP022583) at https://www.ncbi.nlm.nih.gov/sra [54].

As the Piao chicken breed has been maintained for a long time with conservation of its rumpless phenotype, it is difficult to obtain many normal-tailed Piao chicken samples that are not inbred. Since the divergence time between domestic chickens and their ancestor — RJF is short (about 8000 years) [55], we used various normal-tailed chicken breeds as controls. Nevertheless, the number of each control breed was kept small. This design was based on previous studies [56], [57]. In our opinion, the complex constitution of controls would reduce the background noise from specific control breeds and highlight the signatures of the common target trait. Thus, a normal tail is the main feature of controls when compared with the Piao chicken. Besides, the use of exotic breeds as controls should weaken population differences among the Piao chickens, but highlight the common traits in this breed, such as rumplessness.

### Detection of SNPs and indels

Variant calling followed a general BWA/GATK pipeline. Low-quality data were first trimmed using the software Btrim [58]. Filtered reads were mapped to the galGal4 reference genome based on the BWA-MEM algorithm [59], with default settings and marking shorter split hits as secondary. We utilized the Picard toolkit (picard-tools-1.56; http://broadinstitute.github.io/picard/) to sort bam files and mark duplicates with the SortSam and MarkDuplicates tools, respectively. The Genome Analysis Toolkit (GATK; v2.6-4-g3e5ff60) [60] was employed for preprocessing and SNP/indel calling. SNP variants were filtered using the VariantFiltration tool with the options ‘QUAL < 40.0 MQ < 25.0 MQ0 >= 4 && ((MQ0/(1.0*DP)) > 0.1)’. The same criteria were used for filtering indels except settings ‘QD < 2.0 || FS > 200.0 || InbreedingCoeff < –0.8 || ReadPosRankSum < –20.0’.

### Population differentiation evaluation

To estimate population structure, we built a phylogenic tree based on the weighted neighbor-joining method [61] and visualized it using the MEGA6 software [62]. We pruned the SNPs based on linkage disequilibrium utilizing the PLINK tool (v1.90b3w) [63] with the options of ‘--indep-pairwise 50 10 0.2 --maf 0.05’. PCA was performed using the program GCTA (v1.25.2) [64] without GJF. Following the published formulas, we then computed *F*_ST_ [65] and *Pi* [66] values to detect signals of positive selection [56], [66]. A 40-kb sliding window analysis with steps of 20 kb was performed for both *F*_ST_ and *Pi*. Nucleotide diversity (Δ*Pi*) was calculated based on the formula Δ*Pi* = *Pi* (control) – *Pi* (Piao). The intersecting 40-kb SWRs in the top 1% of descending *F*_ST_ and Δ*Pi* were regarded as potentially selected candidates, an empirical threshold used previously [56]. Manhattan plots were drawn using the ‘gap’ package in R [67] ignoring small chromosomes. To reduce false positive rates, we rechecked the candidates by analyzing 1000 replicates of 20 controls randomly sampled from the 98 control chickens. We identified intersecting 40-kb SWRs in the top 1% of descending *F*_ST_ and Δ*Pi* for each random sample. We then checked how many times the candidates were recovered by these intersecting 40-kb SWRs during the 1000 random samples. We calculated the RR (*i.e.*, recovery times divided by 1000) for each candidate and defined those with RR ≥ 0.95 as the final strongly selected ones.

GFs between the Piao and control chickens were compared to retrieve highly differentiated SNPs and indels. In general, there are three genotypes, *i.e.*, 00, 01, and 11. “00” represents that the two alleles are both the same as the reference genome; “01” represents that one of the two alleles is altered, while the other is the same as the reference genome; “11” represents that the two alleles are both altered. Considering sequencing errors, we defined a highly differentiated site using empirical thresholds: first, a site must exist in more than 15 (75%) Piao chickens and more than 50 (51%) control chickens; then, for the eligible site, the ratio with altered alleles must be > 0.8 in Piao chickens and < 0.06 in control chickens. In total, we identified 488 and 48 highly differentiated SNPs and indels, respectively.

### Embryo microscopic observation

Eggs of fertilized Piao and normal-tailed control chickens were purchased from the Zhenyuan conservation farm of Piao chicken and the Yunnan Agricultural University farm, respectively. Eggs were incubated at 37.5 °C with 65% humidity. Embryo tail development was observed from D4 to D10 using a stereoscopic microscope.

### RNA isolation and sequencing

Posterior end samples from a total of 21 chicken embryos (9 Piao and 12 control chickens) were collected from D7 to D9 and stored in RNA*later* at −80 °C. RNA was extracted using Trizol Reagent (Catalog No. 15596026, Invitrogen, Carlsbad, CA) and RNeasy Mini Kit (Catalog No. 74104, Qiagen, Germantown, MD) and purified with magnetic oligo-dT beads for mRNA library construction. Paired-end libraries were prepared by the NEBNext Ultra RNA Library Prep Kit for Illumina (Catalog No. E7530, NEB) and sequenced on the Illumina HiSeq2500 platform after quantification. 150-bp paired-end reads were generated. Overall, we obtained approximately 5 Gigabases of raw data for each library.

For Piao chickens, there were three biological replicates for each of the three developmental days. By using samples from different developmental stages, we aimed to exclude inconsistent effects during development. Due to sampling difficulty, we used 2 Chinese native breeds (6 Gushi and 6 Wuding chickens; Figure S1B) as controls to minimize bias like the genomic analysis. Both control breeds had two biological replicates for each developmental stage. The Gushi and Wuding chickens are native to Henan and Yunnan, respectively, and have normal tails. In our opinion, this approach would strengthen signals associated with the tail when the controls were compared to Piao chickens.

### Transcriptomic data processing

We first trimmed low-quality sequence data using Btrim [58]. Filtered reads were aligned to the reference genome using TopHat2 (v2.0.14) [68], with the parameters ‘--read-mismatches’, ‘--read-edit-dist’, and ‘--read-gap-length’ set to no more than three bases. We evaluated gene expression levels by HTSeq (v0.6.0) with the union exon model and the whole gene model [69], coupled with the Cufflinks program available in the Cufflinks tool suite (v2.2.1) [70] using default parameters.

### Correction and normalization

To improve the analyses, genes were filtered for their expression in three datasets: in at least 80% of the Piao or control samples, gene counts from both HTSeq models (union exon and whole gene) were ≥ 10, while lower bound fragments per kilobase of exon model per million reads mapped (FPKM) values from Cufflinks were > 0. We then performed normalization for gene length and GC content using the ‘cqn’ R package (v1.16.0) [71], based on the filtered count matrix of the HTSeq union exon model. The output values were defined as log_2_ normalized FPKM. The normalized matrix with genes kept in all three datasets was adjusted for unwanted biological and technical covariates, like development days, breeds, and sequencing lanes, via a linear mixed-effects model as previously described [72]. In detail, we calculated coefficients for these covariates with a linear model and then removed the variability contributing to them from the original log_2_ normalized FPKM values. For example, when development days were adjusted, the numbers 1, 2, and 3 were used to replace D7, D8, and D9, respectively. We then calculated a coefficient for each gene using the ‘lm’ function in R, with the number substitutes as a covariate. We removed the product of the coefficient and the number substitute from the log_2_ normalized FPKM value to obtain the adjusted value. The adjusted data were then used for co-expression network construction.

### Differential expression analysis

We applied three methods to identify DEGs. First, 826 DEGs (FDR < 0.05) were identified by the Cuffdiff program in the Cufflinks tool suite with default parameters, using the bam files from TopHat2. Second, 1451 DEGs (FDR < 0.05) were found by DESeq2 [73] based on the read count data from the HTSeq union exon model. Third, 1244 DEGs (FDR < 0.05) were obtained using a linear model based on the log_2_ normalized FPKM matrix, with development stages, chicken breeds, and sequencing lanes as covariates. In total, 437 DEGs found by all three methods were used as the final DEGs.

### Gene co-expression network analysis

To unravel the underlying functional processes and genes associated with tail development, we carried out WGCNA in the R package [40] with a one-step automatic and ‘signed’ network type. The soft thresholding power option was set to 12 based on the scale-free topology model, where topology fit index R^2^ was first greater than 0.8. The minimum module size was limited to 30. A height cut of 0.25 was chosen to merge highly co-expressed modules (*i.e.*, correlation > 0.75). Finally, we obtained a total of 12 modules. M0 consisted of genes that were not included in any other modules, and thus was excluded from further analyses. We performed Pearson correlation analysis to assess module relationships to the rumpless trait, and defined *P* value < 0.05 as the significant threshold.

### Hub genes and network visualization

In general, genes, which have significant correlations to others and the targeted trait, are the most biologically meaningful and thus defined as “hub genes”. Here, we referred to module eigengenes (MEs) as “hub genes”, dependent on high intramodular connectivity values, absolute values of gene significance (GS) greater than 0.2, and absolute values of module membership (kME) greater than 0.8. GS values reflect tight connections between genes and the targeted trait, while kME mirrors eigengene-based connectivity between a gene expression profile and ME, and is also known as module membership [40]. The intramodular connectivity values measure the co-expression degree of a gene to other MEs in the module where it belongs. To visualize a weighted network, we ranked hub genes for each module by intramodular connectivity in descending order, and exported network connections between the top 50 hubs into an edge file with a topological overlap threshold of 0.1. The edge files were input into Cytoscape [74] for network analysis. Network plots for modules significantly related to rumplessness were then displayed based on decreasing degree values.

## Ethical statement

All animals were handled following the animal experimentation guidelines and regulations of the Kunming Institute of Zoology, Chinese Academy of Sciences, China. This research was approved by the Institutional Animal Care and Use Committee of the Kunming Institute of Zoology, Chinese Academy of Sciences, China.

## Code availability

Codes and input files for the major analytic processes were stored in GitHub at https://github.com/WanYMEN/IntegratingGenomicAndTranscriptomicDataToRevealGeneticMechanismsUnderlyingPiaoChickenRumplessTrait.

## Data availability

Raw sequencing data for the RNA samples and DNA samples of Piao chicken reported in this study were deposited in the Genome Sequence Archive [75] at the National Genomics Data Center, Beijing Institute of Genomics, Chinese Academy of Sciences / China National Center for Bioinformation (GSA: CRA001387) which are publicly accessible at https://ngdc.cncb.ac.cn/gsa.

## CRediT author statement

**Yun-Mei Wang:** Conceptualization, Formal analysis, Investigation, Data curation, Writing - original draft, Writing - review & editing, Visualization. **Saber Khederzadeh:** Investigation, Writing - review & editing, Visualization. **Shi-Rong Li:** Investigation. **Newton Otieno Otecko:** Writing - review & editing. **David M. Irwin:** Writing - review & editing. **Mukesh Thakur:** Writing - review & editing. **Xiao-Die Ren:** Data curation. **Ming-Shan Wang:** Conceptualization, Formal analysis, Writing - review & editing, Funding acquisition. **Dong-Dong Wu:** Conceptualization, Resources, Writing - original draft, Writing - review & editing, Supervision, Funding acquisition. **Ya-Ping Zhang:** Conceptualization, Resources, Supervision, Funding acquisition. All authors have read and approved the final manuscript.

## Competing interests

The authors have declared that they have no competing interests.

## Supplementary material

Supplementary Figure S1 **Figure S1 Expression levels of ENSGALG00000013155 and chicken pictures A.** FPKM values of ENSGALG00000013155 in different chicken tissues and development stages from the three NCBI projects (SRA Accessions: ERP003988, SRP007412, and DRP000595) that were used in our previous work [16]. FPKM, the fragments per kilobase of exon model per million reads mapped. **B.** Pictures of the Piao, Gushi, and Wuding chicken. M, male; F, female.

Supplementary Table S1 **RNA and DNA sample information**

Supplementary Table S2 **Strongly selected regions, highly differentiated SNPs and indels, as well as QTL annotations**

Supplementary Table S3 **Expression values of DEGs and their functional annotations**

Supplementary Table S4 **Hub genes of modules significantly correlated to rumplessness**
