# Supplementary figures and images for: Integrating Genomic and Transcriptomic Data to Reveal Genetic Mechanisms Underlying Piao Chicken Rumpless Trait
Source: Genomics Proteomics Bioinformatics. 2021 Feb 23;19(5):787–99. doi: 10.1016/j.gpb.2020.06.019 (PMC9170765; doi:10.1016/j.gpb.2020.06.019)

**A****ERP003988**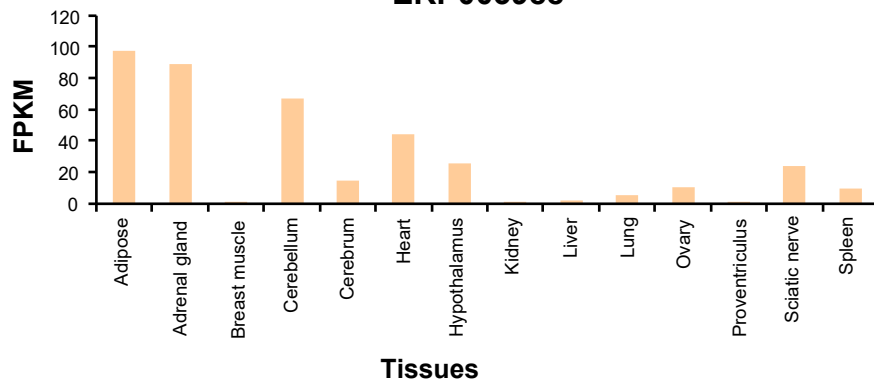**SRP007412**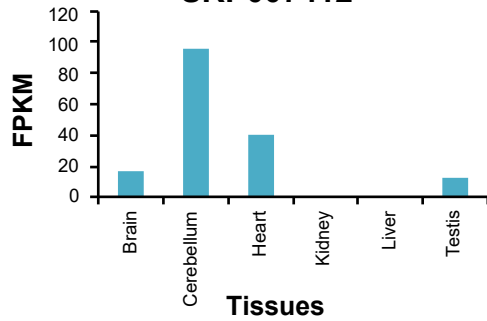**DRP000595**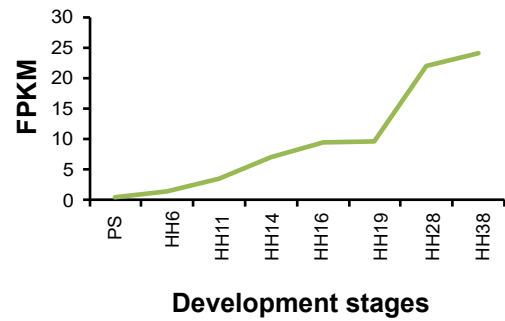**B**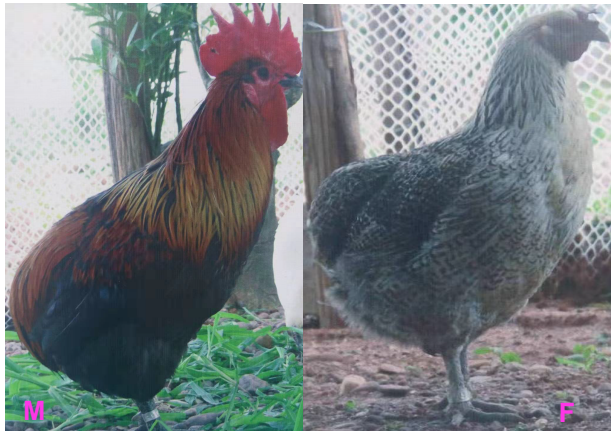**Piao chicken**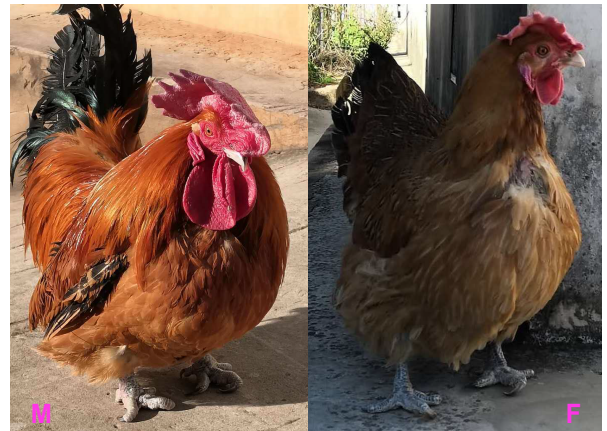**Gushi chicken**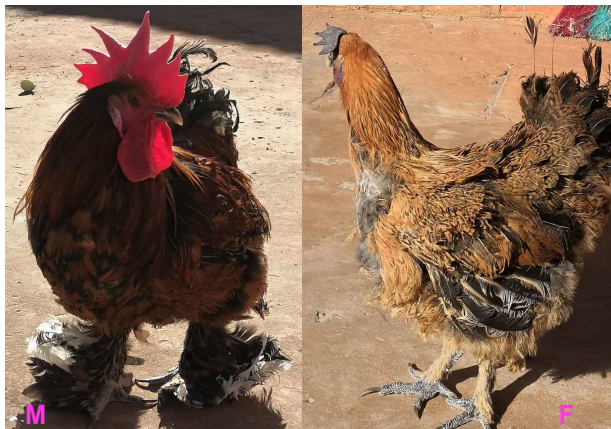**Wuding chicken**

Supplement: Supplementary Figure S1 — Figure S1 Expression levels of ENSGALG00000013155 and chicken pictures A. FPKM values of ENSGALG00000013155 in different chicken tissues and development stages from the three NCBI projects (SRA Accessions: ERP003988, SRP007412, and DRP000595) that were used in our previous work [16]. FPKM, the fragments per kilobase of exon model per million reads mapped. B. Pictures of the Piao, Gushi, and Wuding chicken. M, male; F, female. [file mmc1.pdf]
